# Improving cardiovascular health and quality of life in people with severe mental illness: study protocol for a randomised controlled trial

**DOI:** 10.1186/s13063-018-2748-7

**Published:** 2018-07-11

**Authors:** Malcolm Battersby, Michael R. Kidd, Julio Licinio, Philip Aylward, Amanda Baker, Julie Ratcliffe, Stephen Quinn, David J. Castle, Sara Zabeen, A. Kate Fairweather-Schmidt, Sharon Lawn

**Affiliations:** 1 Mental Health Services, Southern Adelaide Local Health Network (SAHLN), Margaret Tobin Centre, Bedford Park, South Australia 5042 Australia; 2 Department of Family & Community Medicine, University of Toronto, 500 University Avenue, Toronto, ON M5G 1V7 Canada; 3 Global Primary Care, Southgate Institute for Health, Society and Equity, Flinders University, GPO Box 2100, Adelaide, South Australia 5001 Australia; 4 South Australian Health and Medical Research Institute, North Terrace, Adelaide, South Australia 5000 Australia; 5 Division of Medicine, Cardiac and Critical Care Services, Southern Adelaide Local Health Network (SALHN), Flinders Cardiac Clinic, Flinders Private Hospital, Bedford Park, South Australia 5042 Australia; 6 NHMRC Centre of Research Excellence in Mental Health and Substance Use, NHMRC Senior Research Fellow, School of Medicine and Public Health, University of Newcastle, Callaghan, NSW 2308 Australia; 7 Health Economics in the Institute for Choice, School of Business, University of South Australia, City West Campus (WL3-65), GPO Box 2471, Adelaide, South Australia 5001 Australia; 8 Department of Statistics, Data Science and Epidemiology, Swinburne University of Technology, ATC-922, John Street, Hawthorn, VIC 3122 Australia; 9 St. Vincent’s Hospital Melbourne and The University of Melbourne, PO Box 2900, Fitzroy, VIC 3065 Australia; 10 Flinders Human Behaviour & Health Research Unit (FHBHRU), Discipline of Psychiatry, College of Medicine and Public Health, Flinders University, Margaret Tobin Centre, GPO Box 2100, Adelaide, South Australia 5001 Australia

**Keywords:** Flinders Program, Chronic condition self-management, Chronic care model, Cardiovascular disease, Mental illness, Health intervention, Randomised controlled trial

## Abstract

**Background:**

The estimated 300,000 adults in Australia with severe mental illness (SMI) have markedly reduced life expectancy compared to the general population, mainly due to physical health comorbidities. Cardiovascular disease (CVD) is the commonest cause of early death and people with SMI have high rates of most modifiable risk factors, with associated quality of life (QoL) reduction. High blood pressure, smoking, dyslipidaemia, diabetes and obesity are major modifiable CVD risk factors. Poor delivery of recommended monitoring and risk reduction is a national and international problem. Therefore, effective preventive interventions to safeguard and support physical health are urgently needed in this population.

**Methods:**

This trial used a rigorous process, including extensive piloting, to develop an intervention that delivers recommended physical health care to reduce CVD risk and improve QoL for people with SMI. Components of this intervention are integrated using the Flinders Program of chronic condition management (CCM) which is a comprehensive psychosocial care planning approach that places the patient at the centre of their care, and focuses on building their self-management capacity within a collaborative approach, therefore providing a recovery-oriented framework. The primary project aim is to evaluate the effectiveness and health economics of the CCM intervention. The main outcome measures examine CVD risk and quality of life. The second aim is to identify essential components, enablers and barriers at patient, clinical and organisational levels for national, sustained implementation of recommended physical health care delivery to people with SMI. Participants will be recruited from a community-based public psychiatric service.

**Discussion:**

This study constitutes the first large-scale trial, worldwide, using the Flinders Program with this population. By combining a standardised yet flexible motivational process with a targeted set of evidence-based interventions, the chief aim is to reduce CVD risk by 20%. If achieved, this will be a ground-breaking outcome, and the program will be subsequently translated nationwide and abroad. The trial will be of great interest to people with mental illness, family carers, mental health services, governments and primary care providers because the Flinders Program can be delivered in diverse settings by any clinical discipline and supervised peers.

**Trial registration:**

Australian and New Zealand Clinical Trials Registry, ACTRN12617000474358. Registered on 31 March 2017.

**Electronic supplementary material:**

The online version of this article (10.1186/s13063-018-2748-7) contains supplementary material, which is available to authorized users.

## Background

The estimated 300,000 adults in Australia with a severe mental illness (SMI) such as schizophrenia, schizoaffective disorder, bipolar disorder, and depressive psychosis have markedly reduced life expectancy compared to the general population. Importantly, the impact on life expectancy primarily occurs because of physical health comorbidities, where international and local studies show life expectancy is curtailed by 13 to 30 years [1]. Cardiovascular disease (CVD) is common among people with SMI, and is the cause of many early deaths and reduced quality of life [1]. However, a high proportion of this population have readily modifiable CVD risk factors, such as high blood pressure (BP), smoking, dyslipidaemia, diabetes and obesity, where smoking is a key target [2]. A recent survey of Australians (M_age_ = 38 years) with psychotic disorders confirmed their high CVD risk status continues as 76% of those sampled were overweight/obese, had very low physical activity levels, 67% were current smokers, and 47% hypertensive. Analysis of fasting bloods identified that almost 50% had abnormal high density lipoprotein cholesterol and triglyceride levels, and almost 33% had raised glucose [3]. Poor delivery of recommended monitoring and risk reduction is a global problem, again highlighted by a more recent audit [4]. Therefore, effective preventive interventions is urgently needed for people with SMI to support their physical health [5].

However, improving physical health in people with SMI is complicated [6]. Acknowledging this, we adopted the UK Medical Research Council (UK MRC) to help structure the multistep, iterative development and evaluation of our complex intervention [6]. Below, we describe (A) identifying the evidence base, (B) identifying/developing theory, and (C) feasibility stage piloting. Assessment of the intervention using a randomised control trial, rigorous process evaluation, economic evaluation, and contextualising information will underpin the translation of the program into practice.

### Identifying the evidence base

Strategies to improve preventive and physical health management services have focused on either mental and physical health service integration or changing the health behaviours of patients with SMI. Overall, there has been a wide variety of intervention targets and methods, but few studies measure outcomes directly related to CVD risk, and there is little economic evidence of intervention cost effectiveness [5]. Systematic reviews of service integration [7] and cardio-metabolic risk interventions in SMI [8–12], Australian risk reduction guidelines from the National Vascular Disease Prevention Alliance, and reviews of newer technology-based interventions in SMI [13] and mixed populations [14–16], show a need for trials of well-founded clinical systems combined with behaviour change interventions with follow up beyond 12 months [10]. Trials must specifically measure CVD risk among those with SMI and include tailored evidence-supported intervention elements as follows:

1. Health system improvements [7, 10];
2. Tailored interventions for CVD risk-related behaviours [8, 10–12, 17];
  2.1. Tobacco quitting programs with pharmaceutical and structured behavioural support;
  2.2. One-on-one behavioural support for triglyceride reduction and diabetes control;
  2.3. One-on-one behavioural support and group interventions for weight reduction;
  2.4. BP-lowering and lipid-lowering pharmacotherapy according to Australian guidelines;
3. Evidence-informed web-based and mobile technology support options (such as motivational text message prompts) for behaviour change [13–16].

In Australia, Baker et al. [18] recently trialled an intervention comprising nicotine replacement therapy (NRT), information provision, and an intensive programmed group intervention addressing multiple smoking risk-factors. Among smokers with SMI the intervention was no more effective than the control condition comprising NRT, information provision and telephone-delivered attention [18]. Other multiple risk-factor approaches now need to be tested in Australia; approaches which provide a sound clinical system *and* are fully consistent with the above intervention elements.

### Identifying/developing theory

Recovery-based mental health care is a broad approach oriented towards offering patients connectedness, hope and optimism, identity, meaning in life, and empowerment [19]. The approach goes beyond clinical concerns and includes supporting patients to live a fulfilled life. Physical health care that meets patients’ identified needs and goals is therefore an essential part of recovery-oriented care.

The Chronic Care Model describes how the community, health system, delivery system design, decision support, clinical information systems and self-management support combine in the chronic condition management and prevention structure [20]. As self-management is a key, but under-employed requirement of the Chronic Care Model, the Flinders Program [21] specifies a further 12 principles for implementing self-management support in primary care.

The Flinders Program comprises an overarching clinical, person-centred process underpinned by cognitive and behavioural theory and motivational techniques. It integrates self-management support and tailored evidence-based preventive interventions, as identified above, and is consistent with the Chronic Care Model principles of self-management support [22] and recovery-oriented care [23]. Existing research shows sustained behaviour change, in a range of populations, conveys better disease management, improved quality of life [22, 24–28] and preventive health benefits [29, 30].

### Piloting

Service use and pilot studies conducted by the current research team have showed feasibility, high acceptability and good retention in mental health services, and successful use of the Flinders Program in preventive health, justifying progression to a randomised trial for CVD risk reduction in SMI.

There are few existing trials of interventions that reduce absolute cardiovascular risk, rather than individual factors. Further, no effective interventions have been identified for integration into the Australian health care system. For the first time internationally, the Flinders Program will be used to integrate physical and mental health care by engaging patients in their own care through this chronic condition self-management care planning approach. The current trial protocol is presented in accordance with Standard Protocol Items: Recommendations for Interventional Trials (SPIRIT) (see Additional file 1 and Fig. 2).

## Methods/Design

Using the UK MRC guide on Developing and Evaluating Complex Interventions, we piloted the Flinders Program in patients with SMI with the aim of reducing CVD risk and improving quality of life. We now aim to test the intervention in a phase III randomised effectiveness trial (*N* = 358).

**Aim 1** *Objective:* To evaluate the effectiveness and cost benefits of the Flinders Program to reduce absolute CVD risk and improve quality of life in SMI, using a randomised controlled trial design.

**Aim 1** *Hypotheses:* The delivery of the Flinders Program for SMI will be associated with (1) reduced absolute CVD risk, (2) improved quality of life, and (3) will be cost effective.

**Aim 2:** To explore the requirements for a nationally sustainable translation of recommended physical health management in SMI, using qualitative methods which involves all key stakeholders, to develop a policy framework and a comprehensive implementation training package.

### Study design addressing aim 1

A 12-month 2-group randomised (see Fig. 2) controlled trial, with extended follow-up to 24 months will be conducted to contrast the Flinders Program intervention in addition to usual care, with usual care alone.

Each of the two groups will have approximately 178 participants. The intervention group will receive the Flinders Program, in addition to their usual care. Usual care will be provided by health professional care coordinators in public community mental health clinics. Intervention patients and trial nurses will use Flinders Program care planning tools to identify all CVD risk factors, chronic condition and psychosocial issues, patient-identified priority problems and goals, and consensus about which concerns require action.

### The Flinders Program intervention

The Flinders Program provides both framework and tools to engage the patient and structure self-management assessment, tailored planning, motivational enhancement, disease management, prevention, coordination and outcome measurement. It drives optimal use of current health services. In this trial, intervention-allocated participants will be supported to access existing structures and motivational processes including smoking cessation programs; clinical control of triglycerides and diabetes; one-on-one and group components of weight reduction programs; and, where applicable, initiation and adherence with BP-lowering and lipid medications.

Once training has been undertaken, any clinician can deliver the program; however, in the trial’s instance, mental health nurses will facilitate the intervention. Further, while the Flinders Program can be delivered face-to-face, it can also be provided by phone and/or electronically, according to patients’ needs. A web version of the program allows patients and trial nurses to develop the care plan either face-to-face or via the web. This permits communication in real time, or asynchronously via text or email, enabling monitoring and motivational enhancement. In circumstances where participants are randomised into the intervention and subsequently voice issues with continuing to participate in this group, accommodations are made for withdrawal from the intervention program. However, these participants are given the option to continue providing information at the scheduled follow-up data collection points. Details of intervention steps are provided in Table 1.

**Table 1:** Summary of intervention schedule and components

Flinders Program component^a^	Planning and action for:	
	Condition management	CVD risk management
Assessment 1 (week 1) Flinders tools: Partners in Health (PIH), Cue & Response	Patient and trial nurse use Flinders tools to identify all risk factors, chronic condition and psychosocial issues and decide which require action.	Using additional tools and resources: • NVDPA *Guidelines for the Management of Absolute Cardiovascular Disease Risk* • Flinders Program health behaviour assessment tools • Trial nurse database of resources, • Nurse and patient review CVD risk and lifestyle and agree plan for behaviour change among: − Smoking cessation – details below − Diet and exercise – details below − Alcohol use – SA Alcohol and Drug Information Service (ADIS) resources − Lipid-lowering / BP medication via GP
Assessment 2 (week 2) Flinders tools: Problems & Goals (P&G), Flinders Care Plan		
Flinders Care Plan for following 6–12 months shared by trial nurse, patient, psychiatrist, mental health care coordinator and general practitioner (GP).		
Follow-up (week 1–4)	At flexible negotiated follow-up and review contacts (6 in total), the trial nurse: • Monitors outcomes of the care plan using PIH and P&G scores • Assists the patient to achieve goals using motivational and problem-solving approaches and informational and community-based resources • Uses the structured framework of the Flinders Program to coordinate care e.g.,: − as needed, assists access to identified disease specific services e.g. self- management education, home medication review, GP Management Plan and Team Care Arrangements, Chronic Disease Dental Scheme, as per care plan − as needed, assists access to services and coordinates communication between patient and services, social work, Occupational Therapist assessment, Patient Assistance Transport Scheme, financial counselling, local activity groups/courses, Disability Employment Services, Housing, other as per care plan • Reviews and updates Flinders Care Plan as required.	
Follow-up (week 6)		
Follow-up (week 8)		
Follow-up (week 12)		
Follow-up (week 16)		
Follow-up (week 20)		

^a^Assessments face-to-face and follow-ups face-to-face/phone/email/SMS to suit patient needs

### Study design to address aim 2

Concurrent with the trial, views of trial and health service staff, intervention patients, and their family carers will be explored via sequential interviews and focus group discussion (FGD). This will identify enablers and barriers within health systems, practitioner behaviours and patient perceptions, affecting implementation of recommended physical health management in people with SMI.

Twenty patients, 15 family carers and 12 health professionals will be interviewed at different stages of the trial (beginning, middle and end) to gather perceptions of the intervention as a process and outcome. Two FGDs will be also conducted with the health professionals to understand their overall experience at the conclusion of the trial.

### Study setting and study population

The sample population will be patients receiving, or who recently received, treatment and care from southern and western Adelaide community mental health clinics (population of approximately 360,000 patients), including clozapine clinics. Usual care in South Australia, and nationally, comprises monitoring responsibility by dedicated care coordinators trained as mental health professionals (psychiatrists, medical officers, psychiatric nurses, social workers, occupational therapists, and psychologists), referral to general practitioner (GP), if known, and recommendations to access community clinics and programs (all unevenly enacted). We will sample 358 participants in total (across both groups) to allow for an attrition rate of 15%.

### Inclusion and exclusion criteria

Participants will have a diagnosis of schizophrenia, schizoaffective disorder, bipolar disorder, or depressive psychosis established by extant clinical assessment and ICD-10 diagnostic codes; age at least 30 years which allows estimation of absolute CVD risk and matches patient age profile; and at least one CVD risk factor (overweight/obesity, smoking, high blood pressure, blood lipids, glucose or diabetes). However, those without ability to give informed consent; with low English literacy; and/or have acute psychosis or suicidality at baseline will be excluded from participating.

### Recruitment process

Patients will be alerted to the study by their mental health care co-ordinator or a letter from the study team who then will seek (eligible) patients’ permission for a trial nurse to contact them. If permission is granted by the patient, trial nurses will then make contact to provide further details about the RCT and extend an offer to them to participate in the study. Recruitment feasibility is high with 1-month prevalence of psychosis 0.4% in Australia. For example, 250 patients are closely managed for schizophrenia responsive to clozapine in the southern local health network region, with local data showing over 95% meeting risk factor criteria, and two community mental health clinics in this area which currently have 332 (Clinic 1) and 484 (Clinic 2) patients, many with psychoses and CVD risk factors. The western Adelaide health service region also includes large public mental health clinics beyond the southern area from where participants will be recruited if needed.

### Trial procedures

**Fig. 1:**
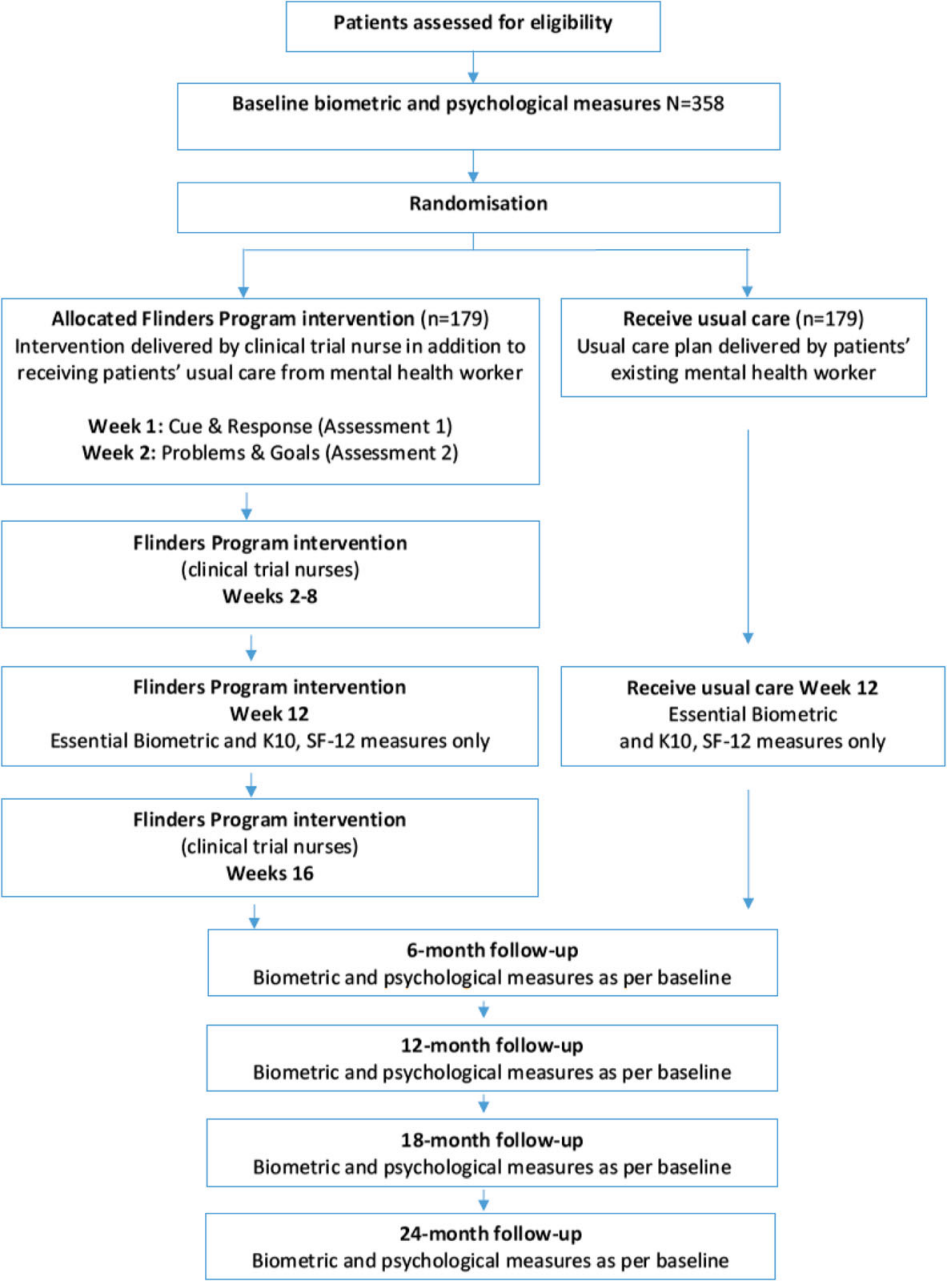
Participant flow across two arms of the RCT

Figure 1 provides a preliminary CONSORT diagram of the study and participant flow. Baseline and follow-up psychometric and biometric data from all patients will be obtained by trial nurses at two main community mental health clinics. However, it is expected that a small number of participants may not be physically able to travel to health clinics; if this should eventuate, trial nurses will make alternative arrangements to facilitate collection of participants’ data. Blood will be taken by trial nurses (trained in phlebotomy) and processed by a community health pathology provider or a Point of Care analysis machine (Roche *cobas® b 101*) able to provide on-the-spot blood results for total and HDL cholesterol, and a glucometer will provide blood glucose levels. Body mass index-based risk scoring will be used if bloods are unattainable. Digital scales and a stadiometer were to used in order to calculate BMI, while a Bedfont *piCO™ Smokerlyzer* will be used to determine the proportion of carboxyhemoglobin (%COHb) and blood carbon monoxide concentration in parts per million (COppm). All data from biometric and psycho-social measures will be entered into the survey application, *Qualtrics,* on a tablet. Full survey data will also backed-up by downloading weekly into a password secured, university file folder.

Post baseline, key patients’ details (Study ID, age, gender) will be sent to an independent clinical trials allocations unit who will be provided with computer-generated protocol for block randomising (i.e., stratified by age and gender) participants. Those randomised into the intervention arm will have their intervention delivered by trial nurses trained in the Flinders Program and study-specific interventions, as illustrated in Table 1. Once the initial Flinders Program assessments have been delivered (Cue & Response; Problems and Goals; see Additional file 2: Table S1), trial nurses will negotiate with participants whether contact is undertaken face-to-face or remotely (e.g., phone; email) on a case by case basis.

Expert Flinders Program trainers will monitor trial nurses by sampling care plans and will provide group and individual supervision initially weekly then monthly to maintain fidelity. Trial nurses will not provide services to ‘control’ group patients. For both groups, other services will continue as usual. However, if at any stage during the intervention, a given participant’s mental health declines to a level that warrants inpatient admission, or where they indicate that they do not wish to continue, s/he will be withdrawn from the trial without adverse impact on their usual care or relationship with the mental health services. The participant can elect to rejoin the trial once the problem is addressed. Post trial, data will be de-identified, and securely stored in a password protected university data repository. Subsequent requests for access to trial data will be considered and then approved/not approved by study CIs.

### Randomisation and blinding

Consenting patients will be randomly assigned to intervention or control with 1:1 allocation ratio. Blocked randomisation within strata (median age, gender) will balance potential confounders and keep group sizes equal, and will use a standard permutated block algorithm to protect concealment. A statistician will independently generate random sequences for each stratum and provide this to a clinical trials allocations unit, independent from the research group, who will randomly allocate participants to intervention or control arm post baseline data collection. Patients cannot be blinded to receipt of the intervention but study information will present the equipoise position. The statistician and health economist will be blinded for comparison of the data sets. Once the final results have been obtained, unblinding is permissible to be able to report and disseminate the trial outcomes.

### Outcomes measures

**Fig. 2:**
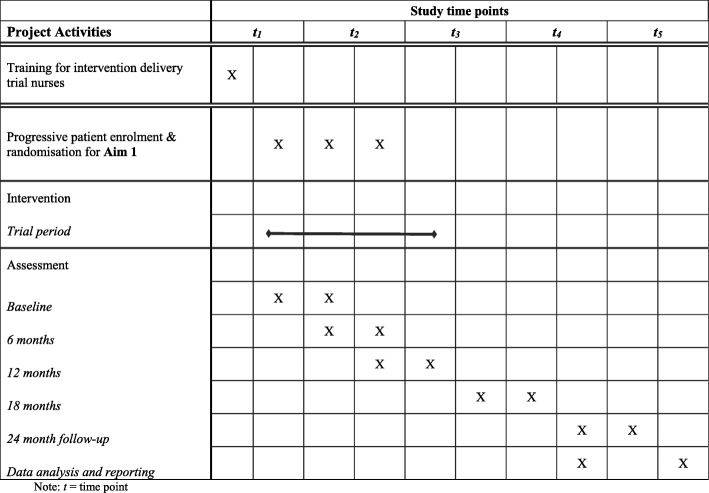
Standard Protocol Items: Recommendations for Interventional Trials (SPIRIT) Figure of enrolment, treatment and assessments over time

As shown in Fig. 2, outcome assessment at baseline, 6,12, 18 and 24 months: bloods and self-report outcomes will be collected by trial staff. Biometric measures constitute total and high cholesterol lipids, blood pressure, weight in kilogrammes, blood glucose level, height and waist measurement in centimetres, %COHb, blood COppm, which constitute the necessary information to calculate the General CVD Risk Score [31]. The self-report measures comprise quality of life [32] SF-12; psychological distress [33] K-10; the Alcohol Use Disorders Identification Test - consumption [34] AUDIT C-3; the Fagerström Test for Nicotine Dependence [35]; the Medical Outcomes Study Sleep measure [36] MOS Sleep; International Physical Activity Questionnaire [37] IPAQ; Australian dietary guidelines healthy eating quiz [38], Partners in Health [39, 40] PIH; Patient Assessment of Chronic Illness Care [41] PACIC; Singh O’Brien Level of Engagement Scale [42] SOLES. Hospital admissions will be extracted from service records to minimise burden on patients and health care staff.

Key outcomes will involve assessing change at 6, 12, 18 and 24 months for the following measures:

1. Absolute CVD risk will be measured using the General CVD Risk Score [31]. This measure applies from age 30 years and includes gender, age, systolic blood pressure, smoking in last week (Y/N), diabetes (Y/N), hypertensive medication (Y/N), and either total and HDL cholesterol or body mass index (Aim 1, Hypothesis 1).
2. Health-related quality of life will be measured by the SF-12 [32], a validated and commonly used instrument, producing summary scores for mental health and physical health components. The Australian SF-6D scoring algorithm developed by Prof Norman and colleagues will be applied to generate utilities and calculate quality adjusted life years [43], (Aim 1, Hypotheses 2 and 3).

### Statistical analysis

Data analyses will be undertaken using an *intention-to-treat* methodology, adhering to CONSORT guidelines. However, where necessary, analyses will also adopt a *per protocol* approach. The following processes have informed the sample size calculation for the 10-year CVD risk change score. Our pilot data (*n* = 17) found mean (SD) of 10-year risk to be 16.9% (9.8%). Thus, taking a conservative baseline mean CVD risk score of 10.5% and a proportionate SD of 6% and assuming a correlation of 0.5 between baseline and follow up scores, a sample of 304 (i.e., 152 per group), provides 80% power to detect a relative change of 20% in 10-year CVD risk. This calculation was achieved using Stata’s *sampsi* command. These changes are realistic; other current empirically-designed interventions providing coordination alone [44, 45] or self-management alone [46, 47] have achieved substantial levels of change. If necessary, we will transform non-normal data to meet normality conditions. The present intervention provides *both* coordination and self-management, and also includes management of CVD risk. We will sample a total of 358 participants allowing for an attrition rate of 15%. Linear regression will be employed to analyse primary outcomes, clustering over nurse delivering the intervention to account for possible correlated outcome measures. The dependent variable will be the outcome at follow-up. The independent variables will be the outcome at baseline and group assignment. Diagnostic tests to examine homoscedasticity and normality of residuals will be carried out to ensure model validity. The calculations for each primary outcome assume a Type I error rate of 2.5% to protect the overall Type I error rate of 5%. A three level random effects mixed models, using Stata’s *mixed* command will be used to analyse outcomes at several timepoints. If necessary, time will be reparametrised. Nurse, and individual within nurse, will be entered as random effects to account for correlated measures within an individual. The dependent variable will be the outcome at each timepoint. The independent variables will be group assignment, time and the product term group ‘x’ time. Differences between groups across time will be assessed via the interaction term. All results will be reported with 95% confidence intervals, and a *p*-value of less than 0.05 (two-tailed) will be deemed to be statistically significant. All analyses will use Stata (StataCorp, College Station, Texas).

### Data and trial monitoring

The study team responsible for the day to day running of the RCT comprise a trial manager, two clinical trial nurses, an administrate support worker, and a PhD student.

A study management committee, chaired by the project lead, with participation from study team members and consumer representatives will meet approximately 3-monthly to oversee the study and ensure effective data management. The committee will also report any adverse event to the South Australian Clinical Human Research Ethics Committee following standard procedures. They will also monitor interim analyses conducted by the statistician and will make the final decision regarding the appropriate stage to terminate the trial.

A national advisory panel of mental health and cardiovascular health experts, and national consumer advocates will meet 6-monthly to provide high level advice to the research group. A stakeholder working group of service managers, psychiatrists, GPs, care coordinators, other clinicians, and consumer advocates with lived experience of mental illness and physical health problems will confirm pathways and agree clinical protocols for mental health, primary care and community providers to ensure sustainability, execution and ongoing communication during the trial. Patients and carers will also be consulted through the Southern Adelaide Mental Health Consumer and Carer Advisory Group.

## Discussion

### Translation: Policy and practice

The outcomes of the study will inform policy and strategy for government and mental health services concerning implementation of recovery-based physical health programs for people with SMI. Clinical academics and researchers within this study team from Victoria, New South Wales, South and West Australia, and policy drivers will form a National Translation Advisory committee which will take account of state-based issues in implementation.

Findings will provide evidence to address the needs of people with SMI and more closely align the National Chronic Disease Strategy self-management and integration action areas and the National Mental Health Plan. Integration of qualitative and quantitative findings on underlying implementation barriers and facilitators will underpin an empirically and theoretically based multi-mode training program for translation at patient, clinical and organisational levels. Existing national training networks will provide a train-the-trainer model to deliver skills training to staff in mental health services as well as on-line and face-to-face implementation training and support for dissemination of the new model of care.

### Expected outcomes and significance

This project aims to address the NHMRC’s Call to Action to build the evidence base and translation elements required to reduce the 13 to 30-year mortality excess experienced by people with SMI, and improve their quality of life. Most deaths in this group are from chronic physical illnesses, especially cardiovascular disease. Despite the availability of clear guidelines for identifying and managing physical health risk factors, they are nonetheless poorly implemented. The problem lies at the disjuncture between mental and physical illness. Mental health services do not routinely focus on the physical health of their patients, partly because they do not have clinical systems to integrate physical and mental health; so too is the case for primary care. Similarly, patients have limited access to services and motivational interventions that focus on addressing physical health in the context of complex psychosocial issues. Our research team has used a rigorous process, including extensive piloting, to develop an intervention which delivers recommended physical health care to reduce cardiovascular risk and improve quality of life. Components of this intervention are integrated using the Flinders Program of chronic condition management which places the patient at the centre of their care and provides a recovery-oriented framework.

### Practical challenges

The trial will adopt a pragmatic and flexible approach, essential when conducting research in the public mental health care sector. In order to counter potential risks to achieving study goals, including low participation rates [48, 49] we have recruited highly skilled and experienced trial nurses, who previously held roles working first hand with the target population. Further, there is a large population of potential participants for the trial to access. We note from previous studies that recruitment issues are commonplace among populations with severe mental illness, particularly with psychotic features, as they are typically difficult to engage. Studies of this type are vulnerable to power-related problems as ultimately they rely on agreement by the patient to participate in the trial.

Another aspect of the trial is that it will operate within a community health context whereby referral of potential participants will be undertaken by community health staff who will be simultaneously undertaking their day-to-day roles; these tasks may be prioritised above the needs of the trial. To lessen the risk to study referral numbers, trial nurses (who are community health employees) will actively facilitating the referral process by maintaining a close relationship with consultant psychiatrists, clinical and care co-ordinators. Additionally, for the trial to effectively evaluate the self-management intervention, the protocol requires a series of longer-term follow-up data collection points represented by *both* care-as-usual (control) and the Flinders Program plus care-as-usual (intervention) participants. We have put in place number of strategies to mitigate dropout, including inviting and reviewing participants’ explanations for initial non-participation and subsequent barriers/challenges to involvement reported by current participants, and non-cash honorariums.

### Summary

By combining a standardised, yet flexible motivational process with a targeted set of evidence-based interventions, the study aims to reduce CVD risk by 20%. If achieved, this ground breaking outcome will be translated nationally and internationally. It will be of great interest to people with mental illness, carers, mental health services, governments and primary care owing to the capacity for the Flinders Program to be delivered within all of these settings by any clinical discipline and supervised peers. Project outcomes will be published in scientific journals, reports and presentations in order to disseminate the findings which are underpinned by a best-practice CVD prevention intervention designed specifically for people with SMI. Further, a training package for health services could be developed so that physical health interventions can be broadly, successfully and sustainably implemented in populations with SMI. This study’s results have the capacity to inform policy and promote closer alignment of the National Chronic Disease Strategy and the National Mental Health Plan. These data are anticipated to constitute a rich resource, and investigators are keen to follow the cohort over the next decade and beyond.

## Trial status

Protocol Version 1; Date: 15.02.2018.

Recruitment commenced in November 2017 and will be completed December 2018.

## Additional files

Additional file 1: SPIRIT 2013 Checklist: Recommended items to address in a clinical trial protocol and related documents*. (DOC 125 kb)

Additional file 2: **Table S1.** The Flinders Program Tools. (DOCX 16 kb)

## Abbreviations

CCM: Chronic condition management
CVD: Cardiovascular disease
FGD: Focus group discussion
GP: General Practitioner
NHMRC: National Health and Medical Research Council
NRT: Nicotine Replacement Therapy
QoL: Quality of Life
RCT: Randomised Controlled Trial
SALHN: Southern Adelaide Local Health Network
SD: Standard deviation
SMI: Severe mental illness
UK MRC: United Kingdom
