# Supplementary material for: Improving cardiovascular health and quality of life in people with severe mental illness: study protocol for a randomised controlled trial
Source: Trials. 2018 Jul 11;19:366. doi: 10.1186/s13063-018-2748-7 (PMC6042320; doi:10.1186/s13063-018-2748-7)
Supplement: Supplementary file 2 — Table S1. The Flinders Program Tools. (DOCX 16 kb) [file 13063_2018_2748_MOESM2_ESM.docx]

Additional file 2 Table S1. The Flinders Program Tools

| **Component** | **Processes** | **Completion Time** |
| --- | --- | --- |
| 1. Partners in Health Scale (PIH) | These are 12 questions where the patient rates their self-management capabilities. Each question is scored on a 9-point Likert-scale where 0=no self-management and 8=high self-management. | 3-5 minutes |
| 2. Cue and Response Interview | The health professional and the patient have an in-depth conversation about the abovementioned 12 PIH questions in order to establish what the patient knows, what they do well, and the potential enablers and barriers to effective self-management. Each question is then scored by the health professional on a 9-point Likert-scale where 0=no self-management and 8=high self-management. They then compare their score for each question with the patient’s score. Discussion of any discrepancies between their views is undertaken. Problems to be addressed within the care plan are collaboratively determined by the patient and health professional; this step provides key detailed assessment information. | 20-45 minutes |
| 3. Problem and Goal Assessment | The patient’s current priority issue affecting their life and a “SMART” goal, led by the patient are then determined. | 10-20 minutes |
| 4. Self-Management Care Plan | Developed with full collaboration and agreement with the patient, listing priority issues, what the patient wants to achieve, steps to get there, who is responsible and an agreed review date to monitor progress. | 15-20 minutes |
